# Microbial communities during the composting process of *Agaricus subrufescens* and their effects on mushroom agronomic and nutritional qualities

**DOI:** 10.3389/fmicb.2024.1471638

**Published:** 2024-11-15

**Authors:** Chunxia Wang, Dongxia Wang, Chao Li, Zhaopeng Ge, Liubin Hao, Gadah Albasher, Fan Feng, Yue Sun, Yanfen Lyu, Suyue Zheng

**Affiliations:** ^1^College of Landscape and Ecological Engineering, Hebei University of Engineering, Handan, China; ^2^China United Network Communications Co., Ltd. Zhejiang Company, Hangzhou, China; ^3^Chengde Academy of Agricultural and Forestry Sciences, Chengde, China; ^4^Department of Zoology, College of Science, King Saud University, Riyadh, Saudi Arabia; ^5^Department of Mathematics and PhysicsScience and Engineering, Hebei University of Engineering, Handan, China; ^6^College of Veterinary Medicine, South China Agricultural University, Guangzhou, China

**Keywords:** composting, *Agaricus subrufescens*, microbial communities, high-throughput sequencing, metabolic function

## Abstract

**Introduction:**

Tunnel composting technology for preparing *Agaricus subrufescens* cultivation media can achieve a higher biological efficiency (BE) and a lower contamination rate (CR). However, this technology lacks in-depth and systematic study.

**Methods:**

In the present study, the changes in the microbiome and microbial metabolic functions were surveyed using metagenomic analysis. The physicochemical parameters, agronomic properties and nutritional qualities were also evaluated.

**Results and discussion:**

Results showed that the contents of cellulose, hemicellulose and lignin dropped to 10.18, 11.58, 27.53%, respectively at the end of composting. The tunnel composting technology led to significant increases in crude protein content (32.56%) and crude fiber content (13.68%). Variations of physicochemical characteristics led to different successions of microbial communities. Bacteria manifested significantly higher abundance than fungi. Firmicutes, Actinobacteriota, Chloroffexi and Deinococcota were the predominant bacterial phyla. Ascomycota and Basidiomycota were the dominant fungal phyla in the thermophilic phase. *Pseudonocardia*, *Truepera*, and *Thermopolyspora* were positively correlated with the yield of *A. subrufescens*. In addition to TN, most of the physicochemical properties were significantly correlated with fungal communities in the thermophilic phase. The metabolisms of carbohydrate, amino acid and energy were the primary enrichment pathways. These findings deepen the understanding of microbial communities composition during the composting of *A. subrufescens* substrates. Moreover, this study provides a basis for improving tunnel composting technology.

## 1 Introduction

Tunnel composting technology is an effective way to produce edible fungi culture materials on a large scale. The composting site is a scientifically designed tunnel, and the bottom of the tunnel in contact with culture materials is hollow steel plate. This design is not only conducive to the ventilation and exhaust of the culture materials, but also can avoid the water deposition at the bottom of the culture materials, reduce the pollution rate, and significantly improve the composting efficiency (Ardolino et al., 2017). The traditional composting is usually carried out outdoors. Details of the traditional composting process vary between countries, but typically include a wetting and pile building phase, a thermophilic phase (Phase I, 65–70°C), in which the structural components of the raw materials are degraded, and a pasteurization phase (Phase II, 58–62°C), in which the breakdown products are incorporated into microbial biomass (Largeteau et al., 2011; Thai et al., 2022). Compared with traditional composting technology, tunnel composting technology can accurately control the temperature and humidity, so that the temperature and moisture of each region of culture materials are consistent and the composting time is significantly shortened. During the tunnel composting process, machinery is used to turn the pile, which can make the composting quality of culture materials uniform and stable (Meng et al., 2019). At the same time, the tunnel composting technology is not limited by the external environmental conditions, and it can compost normally even in rainy days. Therefore, the quality of the culture materials is better, which is more conducive to the growth of mycelia, so as to ensure the high and stable yield of edible fungi.

*Agaricus subrufescens* is referred to as “Ji Song Rong” in China, “sun mushroom” in Brazil, “almond mushroom” in the USA (Almeida et al., 2022). *A. subrufescens* is not only nutritious and delicious, but also rich in polysaccharides, phenolic acids and ergosterol (Taofiq et al., 2019). Since its discovery in 1893, *A. subrufescens* has been cultivated throughout the world for its medicinal properties and Brazil, China, United States and Japan are the main producers (Almeida et al., 2022; Wisitrassameewong et al., 2012). *A. subrufescens* has been produced at large scale in China since 1990s (Largeteau et al., 2011). In recent years, the yield of *A. subrufescens* has increased year by year. According to the statistics of China Edible Fungi Association (CEFA), the annual output of *A. subrufescens* reached 128,000 tons in 2022. The medicinal mushroom *A. subrufescens* is a typical grass rot fungus and can be cultivated on a variety of raw materials (Rózsa et al., 2019). In Brazil, the raw materials mainly include sugar cane bagasse, cassava fibers and chicken manure. In Europe, the main raw materials are horse or poultry manures and wheat straw. In China, cotton seed hull, rice hull, cow manure and corncob are utilized to cultivate *A. subrufescens* (Largeteau et al., 2011; Llarena-Hernández et al., 2013)*. A. subrufescens* is cultivated on compost by a procedure based on the similar method used to grow *Agaricus bisporus* (Pardo-Giménez et al., 2020). In addition to environmental factors, casing layers and layering styles also play extremely important role in the yield of *A. bisporus* (Adanacioglu et al., 2015).

Composting is a sustainable and attractive method for the cultivation of edible fungi. The composting converts organic matter into small molecules, reduces environmental pollution and requires lower input (Kang et al., 2014; Kong et al., 2020). Short-term composting for oyster mushroom cultivation is well established in China (Liu et al., 2022; Guo et al., 2021; Yang et al., 2022). The substrates for *Volvariella volvacea* and *A. bisporus* need to undergo two composting processes (Kertesz et al., 2016; Vos et al., 2017). The microbial community structures in composting of culture materials have been extensively studied. A range of thermophilic proteobacteria and actinobacteria played an extremely important role in the key process of button mushroom substrate composting (Kertesz and Thai, 2018). During corncob composting for preparation of cultivation medium for *Pleurotus ostreatus*, the changes of physicochemical properties lead to different dominant phylum and Firmicutes was the dominant phylum at thermophilic stage (Kong et al., 2020). Firmicutes, Actinobacteriota and Proteobacteria were the dominant bacterial phyla in short-term peach sawdust-based composting for preparing oyster mushroom cultivation media (Guo et al., 2021). *Streptococcus* and *Lactococcus* were predominant bacterial genera at the beginning stage of composting cultivation of *Pleurotus floridanu*s, while *Acinetobacter* became predominant at the ending stage (Wei et al., 2024).

Different raw materials and cultivation techniques lead to differences in mushroom yields and nutritional properties of fruiting bodies. Tunnel composting technology has been widely applied in the composting for preparation of cultivation medium for *A. subrufescens*, so as to realize the factory cultivation. However, few studies have reported the microbial community succession in the composted substrates. Therefore, this study is aimed to determine the variations of bacterial and fungal communities during the composting process using tunnel composting technology and their interactions with agronomic properties and nutritional qualities. These results will provide theoretical basis for adjusting the technical parameters of tunnel composting technology, so as to further promote the industrial cultivation of *A. subrufescens*.

## 2 Materials and methods

### 2.1 Materials

The raw materials included cow manure, corncob, wheat straw, superphosphate, calcium carbonate, lime, and gesso. Cow manure was purchased from a cattle farm in Quzhou and was thoroughly dried and crushed before use. Other raw materials were obtained from local agricultural material stores (Handan, Hebei Province, China). In addition, corncob and wheat straw were also cut into small particles of 3–4 cm using a grinder. The physicochemical properties of raw materials were showed in Supplementary Table S1.

### 2.2 Composting process and sample collection

During the tunnel composting process, the total dry weight of raw materials for a tunnel was 131 tons, including 49% cow manure, 31% wheat straw, 15% corncob, 0.9% superphosphate, 1.8% gesso, 0.8% calcium carbonate and 1.5% lime. A tunnel of composting substrates could be used to grow 2000 square meters of *A. subrufescens*. The composting process took two periods (phase I and phase II), 15 days and 5 days, respectively. The composting process was as follows: first of all, the raw materials were fully stirred, and then the raw materials were pre-wet by spraying, which took 2 days. Superphosphate, calcium carbonate, gesso and lime were added to the raw materials, which were then transported into the primary composting tunnel by forklift. According to temperature change, the ventilation time and ventilation volume were determined, and the first stack-turning process started every 5 days from the fifth day. After the first composting, the pH value of the culture materials was about 8, and the water content was about 75%. The composting substrates were transferred to the secondary composting tunnel by forklift, and the height of the material pile was 1.2 m. The temperature of culture materials was raised to 60°C for pasteurization for 8–10 h. After pasteurization, the temperature of culture materials dropped to 50°C, at which time the composting continued for 4 h to 5 day. Finally, the temperature was lowered to below 30°C by fresh air. At this time, the composting substrates were transported into the mushroom house to prepare for sowing. The samples were collected at three time points during the entire composting process: C1 (day 0: unfermented), C2 (day 15: first composting completed), C3 (day 20: second composting completed). Each representative sample of equivalent weight was randomly collected from 15 different sites in three depths as follows: 5 sub-samples (upper part of the pile), 5 sub-samples (middle part of the pile), 5 sub-samples (lower part of the pile). The 15 sub-samples were thoroughly mixed by hand and then divided into two parts. The first part was stored at −80°C for metagenomic analysis. The second part was air-dried and sieved to 0.25 mm for physicochemical analysis.

The composting process was different between traditional and tunnel composting. The raw materials of the traditional composting included 60% corn straw, 36% cow manure, 0.9% urea, 1% calcium superphosphate, 1% gypsum powder, 0.4% phosphate fertilizer, 0.7% lime. The corn straw and cow manure were shattered into particles of 15–30 cm and 1–5 cm, respectively, via a grinder. Before composting, the raw materials were humidified with water at a weight ratio of 1:2.5 (raw materials: water). After homogeneous stirring, the raw materials were stacked into a trapezoidal heap with a height of 150 cm, a width of 100 cm at the top and a width of 200 cm at the bottom, the length was not limited. The composting site was flat, well ventilated and clean. When the temperature of composting substrates was raised to 65–70°C, the first turn was conducted. The substrates of the bottom layer and the outer layer were turned to the middle, and the substrates in the middle were turned to the outer layer. After that, the pile was turned every 3–4 days, a total of 3–4 times, and the first composting was completed. When the temperature of the culture materials did not drop, the composting substrates were transported to mushroom house for the second composting. The substrates were evenly spread on the mushroom bed with a thickness of 18–22 cm. The steam was delivered to the mushroom house to raise the temperature to 58–62°C within 1–2 days and maintain for 6–8 h. Then the temperature was cooled to 48–52°C by ventilation and maintained for 5–7 days. At this time, the second composting was completed.

### 2.3 *Agaricus subrufescens* cultivation

Although the two composting processes were different, the cultivation method of *A. subrufescens* was the same. The strain “Fuji77” of *A. subrufescens* was used in this study and stored in the Agricultural Culture Collection of China. The temperature of conditioned compost was adjusted to 28°C by increasing the intake of fresh air. The composted substrates were evenly spread on the mushroom bed with a thickness of 18–22 cm. The spawns were sowed evenly over the surface of the substrates with a rate of 3% (fresh weight, v/v). After sowing, the room temperature was controlled at 25–27°C for mycelial culture. When the mycelia grew to two-thirds of the substrates, a layer of peat soil with a thickness of 3 cm was covered above the mycelia. After 10–12 days of soil covering, primordium induction was performed by increasing aeration, maintaining 85–90% relative humidity and decreasing the temperature. Subsequently, the fruiting management and harvest were carried out. The fruiting bodies were collected for four flushes.

### 2.4 Physicochemical analysis of composting substrate

These physicochemical properties of temperature, moisture content, pH, electrical conductivity (EC), total carbon (TC), and total nitrogen (TN) were measured according to previously reported methods (Guo et al., 2021; Zhang et al., 2019). The contents of cellulose, hemicellulose, and lignin were performed as described by Liu et al. (2022). Assays of laccase activity were performed by ABTS method (Parenti et al., 2013). Assays of cellulase and xylanase activity were performed by DNS method (Zhang et al., 2016). All the experiments were performed in triplicate.

### 2.5 Agronomic and nutritional qualities of fruiting bodies

The fruiting bodies were harvested at mature and the agronomic properties were analyzed, including total yield of four flushes, BE and CR (Wei et al., 2024). The fruiting bodies of first-flush mushrooms were cleaned with distilled water and dried at 55°C until a constant weight was achieved. Next, the dried mushroom samples were pulverized, sifted through a 200-mesh sieve and stored at 4°C for further nutritional quality analysis. The determination of crude fiber content was based on the Chinese National Standard Method (GB/T 5009.10–2003). The crude fat content was calculated based on the Chinese National Standard Method (GB/T 5009.6–2016). The crude protein was obtained using the Chinese National Standard Method (GB/T 5009.5–2016) (Zhang et al., 2019; Jacinto-Azevedo et al., 2021; Guo et al., 2021).

### 2.6 DNA extraction, library preparation, and sequencing

The Fast DNA SPIN Kit for Soil (MP Biomedical, USA) was applied to extract the genomic DNA of all composting samples. The extracted DNA was determined by 1% (w/v) agarose gel electrophoresis. The concentration and purity of DNA were checked using a NanoDrop® ND-2000 spectrophotometer (Thermo Scientific Inc., USA). These checked DNA were used as templates to amplify genes of fungi and bacteria by an ABI GeneAmp® 9,700 PCR thermocycler (ABI, USA). The fungal ITF genes of the ITS1 region were amplified using ITS1F (5′-CTTGGTCATTTAGAGGAAGTAA-3′) and ITS2R (5′-GCTGCGTTCTTCATCGATGC-3′) and the bacterial 16S rRNA genes of the V3-V4 region were amplified with 338F (5′-ACTCCTACGGGAGGCAGCA-3′) and 806R (5′-GGACTACHVGGGTWTCTAAT-3′). The PCR reaction mixture and amplification procedure that we used were previously reported (He et al., 2022). The AxyPrep DNA Gel Extraction Kit (Axygen Biosciences, USA) and Quantus™ Fluorometer (Promega, USA) were used to purify and quantify the PCR products, respectively. Miseq libraries were prepared using TruSeq™ DNA Sample Prep Kit (Illumina, USA). After that, the PCR products were sent to Majorbio Bio-Pharm Technology Co., Ltd. (Shanghai, China) for high-throughput sequencing on an Illumina MiSeq PE300 platform platform.

### 2.7 Metagenomic analysis

Raw metagenomic sequences were merged, trimmed, filtered, and aligned using Trimmomatic version 0.36 and FLASH version 1.2.7 (Bolger et al., 2014). The optimized sequences were clustered into operational taxonomic units (OTUs) with 97% sequence similarity level using UPARSE version 7.1^1^ (Edgar, 2013). In order to obtain the taxonomy of each OTU, the RDP Classifier version 2.2 was used to analyze bacterial and fungal sequences against the SILVA database (Release 132^2^) and the UNITE database (Release 7.2^3^), respectively (Nilsson et al., 2019). Alpha-diversity was calculated to elucidate the variations in the diversity and richness of the microbial communities using Mothur version 1.30.1^4^ (Schloss et al., 2009). The heat map of community was made using R language vegan package. Principal coordinate analysis (PCoA) and non-metric multidimensional scaling (NMDS) were performed on the Majorbio I-Sanger Cloud Platform.^5^ The linear discriminant analysis (LDA) effect size (LEfSe) was applied to identify the significantly abundant bacteria and fungi in different treatments (LDA scores >4.0, *p* < 0.05) The PICRUSt2 was performed to predict the metagenomic function (Douglas et al., 2020).

### 2.8 Statistical analysis

All physicochemical properties of composting substrates were measured in triplicates. Microsoft Excel 2016 and GraphPad Prism version 8.0 were used for all data generation. SPSS version 25.0 was applied to conduct statistical analysis and the significance level of differences was set at *p* < 0.05.

## 3 Results and discussion

### 3.1 Physicochemical characteristics of composting substrate

**Figure 1:**
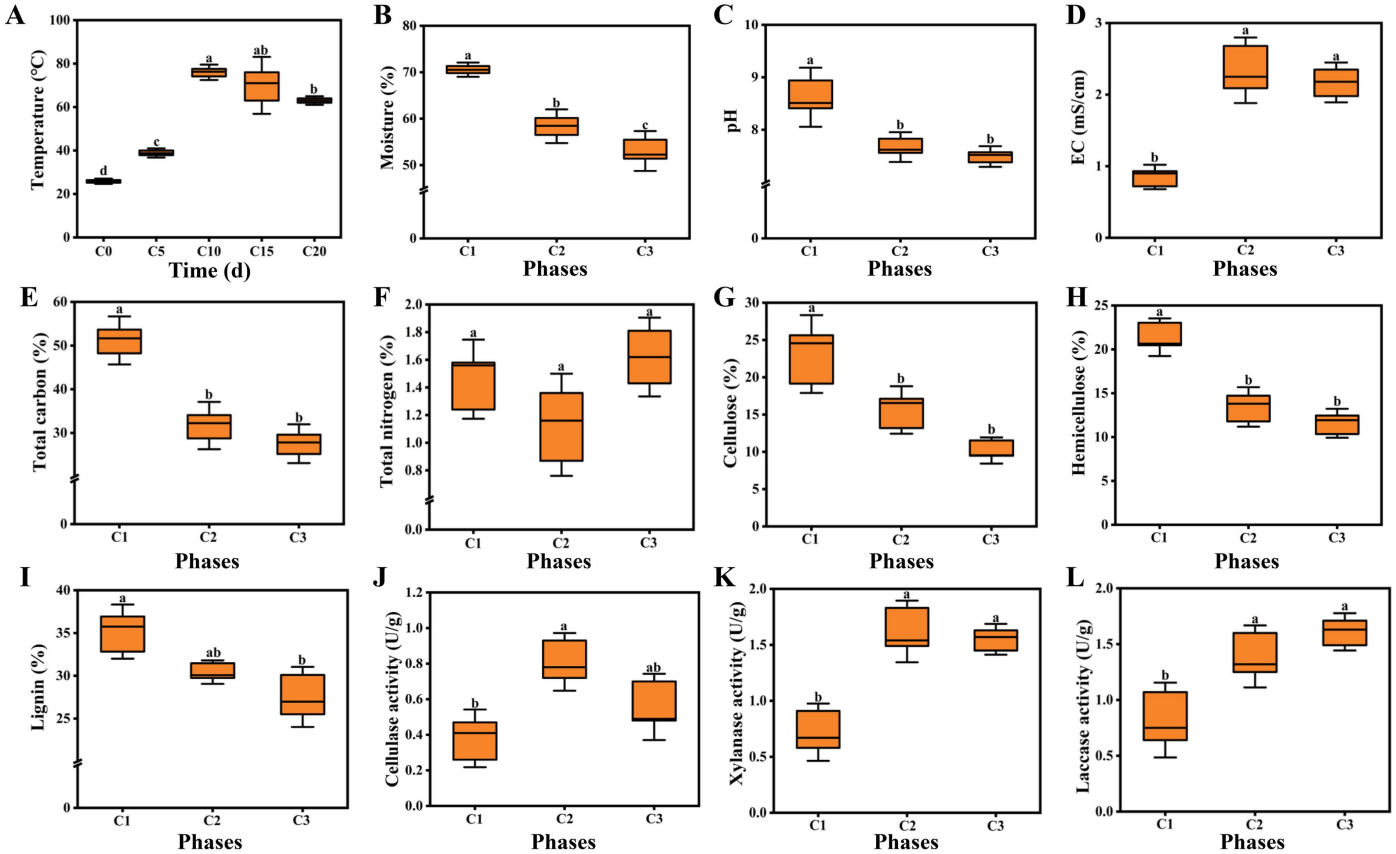
**(A)** Temperature; **(B)** Moisture; **(C)** pH; **(D)** EC; **(E)** Total carbon; **(F)** Total nitrogen; **(G)** Cellulose; **(H)** Hemicellulose; **(I)** Lignin; **(J)** Cellulase activity; **(K)** Xylanase activity; **(L)** Laccase activity. C1, C2, and C3 represented day 0, 15, and 20 in composting, respectively. Data were shown as the mean ± SD of three independent experiments. Different letters indicated significant differences (*p* < 0.05).

Physicochemical properties are important indicators of composting, which can affect the rate of lignocellulose degradation, reflect the succession of microbial community, and even determine the yield and quality of edible fungi (Xu et al., 2020; Zhou et al., 2019). The entire composting process lasted twenty days and the pile temperature was measured every five days. Figure 1A showed the changes of pile temperature. The temperature softly increased during the initial five days (from 25.7°C to 38.9°C). However, the temperature underwent a sharp increase and reached a peak at 76°C on the tenth day. The thermophilic phase (63–76°C) lasted for five days until the end of the first composting. The thermophilic level was slightly lower than that of *A. bisporus* (70–80°C) (Kertesz and Thai, 2018). In the second composting process, the pile temperature dropped slightly, but was still above 60°C. High temperature plays an extremely important role in compost maturity and quality, which can not only kill worm eggs and miscellaneous bacteria, but also convert complex macromolecules into small molecules that are easily absorbed by edible fungi (Joseph et al., 2018; Zhou et al., 2019). Water is an important factor driving the composting. When the moisture content is too low, it is detrimental to the activity of microorganism. On the contrary, it is not conducive to ventilation leading to anaerobic compFosting. The moisture content showed a continuous decreasing trend. During the period from C1 to C2, the moisture content significantly decreased (from 70.56 to 58.37%). The moisture content decreased to 53.04% in C3 phase, but the decrease was smaller than that in C1–C2 phase (Figure 1B). High temperature and microbial metabolism were the main reasons for the decline of moisture content (Guo et al., 2021; Li et al., 2021). As shown in Figure 1C, the pH sharply dropped from the initial 8.62 to 7.67 during phase C1–C2, then softly decreased to 7.49, which was consistent with the finding of a previous study reported by Liu et al. (2022). The final pH was suitable for the cultivation of *A. subrufescens*. The variations in EC were shown in Figure 1D, the EC value of C2 phase was obviously higher than that in the initial phase, which was due to the release of NH_4_^+^ and NO_3_^−^ (Zhang et al., 2020). Then the EC went through a slight decline with the final value of 2.17 mS/cm (EC < 3.00 mS/cm). The EC of *A. bisporus* composting substrates was stable at 2.5–3.0 mS/cm throughout (Thai et al., 2022). Carbon source is the basic material for the survival of microorganism, so the dynamic changes of carbon content can reflect the succession of microorganism (Gao et al., 2023; Wan Mahari et al., 2020). Total carbon accounted for 51.19% in the initial phase of composting and evidently decreased with the composting time. The content of total carbon decreased from 31.69% (C1) to 27.54% (C2) (Figure 1E). As one of the most sensitive indicators of composting, the TN content was crucial to the yield of edible fungi. According to a report by Kong et al. (2020), the TN content ranged from 1.0–2.0% was optimum for mycelial growth. As shown in Figure 1F, the TN content of the original sample was 1.46%. However, it decreased by 22.60 to 1.13% during the thermophilic phase. Similar results were obtained by Thai et al. (2022). It was due to the conversion of NH_4_-N to NH_3_ and subsequently by its volatilization (Wang et al., 2013). The TN content increased to 1.62% in maturation phase (C3), which might result from the reduction in total weight of the composting substrates (Kong et al., 2020; Tong et al., 2019).

The changes of lignocellulose content and degrading enzyme activity were also determined. The contents of cellulose, hemicellulose and lignin all exhibited decreasing trends during the composting following the order: C1 > C2 > C3. The contents reached 10.18, 11.58, 27.53%, respectively at the end of composting (Figures 1G–I). In addition, compared with cellulose and hemicellulose, the degradation rate of lignin was the lowest, which might be due to its water insoluble, irregular and highly branched structure (Ma et al., 2020; Zhong et al., 2021). Cellulase played a central role in cellulose degradation (Guo et al., 2021). Overall, we observed that cellulase activities in C2 and C3 phases were significantly higher (113.16, 47.37%) than that in the initial phase and the highest activity occurred in C2 phase (Figure 1J). Xylanase was the critical enzyme involving in hemicellulose degradation (Kumar et al., 2018; Guo et al., 2021). The activity of xylanase was 0.72 U/g in the initial phase of composting and reached 1.62 U/g in C2 phase. After the thermophilic phase, the activity of xylanase softly decreased to 1.55 U/g, which had no significant difference from that in C2 phase (Figure 1K). This trends was different from previous study of *A. bisporus* compost substrates reported by Thai et al. (2022), which may be caused by different raw materials. As can be seen from Figure 1L, the initial laccase activity was 0.82 U/g. With the advancement of the composting process, laccase activity continuously increased and reached the maximum of 1.61 U/g in C3 phase. Compared with laccase, the highest activity of cellulase and xylanase occurred in early composting phase (C2), which was due to the fact that microorganisms were the first to break down easily degradable cellulose and hemicellulose.

### 3.2 Agronomic and nutritional qualities of fruiting bodies

Nowadays, *A. subrufescens* is cultivated at the industrial level in Brazil, China, Japan, and Korea and cultivated on compost based on two-phase process (Largeteau et al., 2011). The composting process affect the quality of mushroom substrates. Moreover, the composting substrates directly determines the yield and quality of mushrooms. As shown in Table 1, the tunnel composting represented a lower CR (11.34%) compared with the traditional composting (28.65%). The low level of CR led to the high BE and yield. The tunnel composting represented significantly higher BE of 100.26%. The total yield of tunnel composting of five flushes was 10.85 kg, which was significantly higher than those of traditional composting (8.19 kg). Oyster mushrooms cropping on short composting substrates showed a BE range of three flushes 73.91–100.19% (Wei et al., 2024). These results indicated that an appropriate composting method could improve the yield and BE of *A. subrufescens*. In addition, the nutrient compositions in mushrooms changed with the composting process. The tunnel composting led to significant increases in crude protein content (32.56%) and crude fiber content (13.68%), which increased by 18.75 and 30.16%, respectively. The crude protein content and crude fiber content of mushrooms varied greatly depending on cultivation substrates. *Pleurotus floridanus* showed a crude protein content range of 23.3 to 29.8% based on different substrates (Andrew, 2022). Compared with the two composting methods, there was no significant difference in the crude fat content. It showed that the composting process of cultivation substrates could significantly improve the nutritional quality of *A. subrufescens*, but not all the nutrients.

**Table 1:** Agronomic properties and nutritional qualities of fruiting bodies.

Method	Total yield (kg·m^−2^)	Biological efficiency (%)	Contamination rate (%)	Crude protein (%)	Crude fat (%)	Crude fiber (%)
Traditional composting	8.19 ± 0.37b	92.98 ± 0.34b	28.65 ± 3.19a	27.42 ± 1.89b	4.27 ± 0.68a	10.51 ± 0.67b
Tunnel composting	10.85 ± 0.64a	100.26 ± 0.71a	11.34 ± 2.55b	32.56 ± 3.51a	4.51 ± 0.33a	13.68 ± 0.92a

All the data were expressed as means ± SD (*n* = 3). Different lowercase letters indicate significant differences (*p* < 0.05).

### 3.3 Illumina sequencing of composting substrate microbiomes

The metagenomics sequencing of bacterial communities generated 1.9 × 10^8^ raw reads, and each group of samples were 5.39 × 10^7^–7.12 × 10^7^ reads. The clean reads of a single sample after data quality control accounted for more than 99% of the original reads. A total of 458,633 assembly sequences were acquired. The average length was 411–415 bp. While 1.7 × 10^8^ raw reads and 670,316 sequences were obtained during the analysis of fungal communities. The length range of sequences was 141–454 bp (Table 2). The raw 16S rDNA and ITS sequencing reads were uploaded to the NCBI Sequence Read Archive (SRA) database (Accession Number: PRJNA917118, PRJNA884292).

**Table 2:** Information about sequencing of metagenomic DNA samples from the composting substrates.

	Sample	Raw reads	Clean reads	Clean reads rate (%)	Sequence number	Min length (bp)	Max length (bp)	Average length (bp)
Bacteria	C1	53,951,286	52,872,260	99.8	130,000	245	463	415
	C2	71,208,361	69,072,110	99.7	170,542	265	503	417
	C3	64,949,911	63,650,912	99.8	158,121	243	474	411
Fungi	C1	54,510,735	52,875,412	99.7	205,669	141	454	265
	C2	72,823,770	70,639,056	99.7	282,245	174	420	258
	C3	47,229,979	46,285,379	99.8	182,402	168	322	259

### 3.4 Dynamic succession of microbial communities

**Figure 2:**
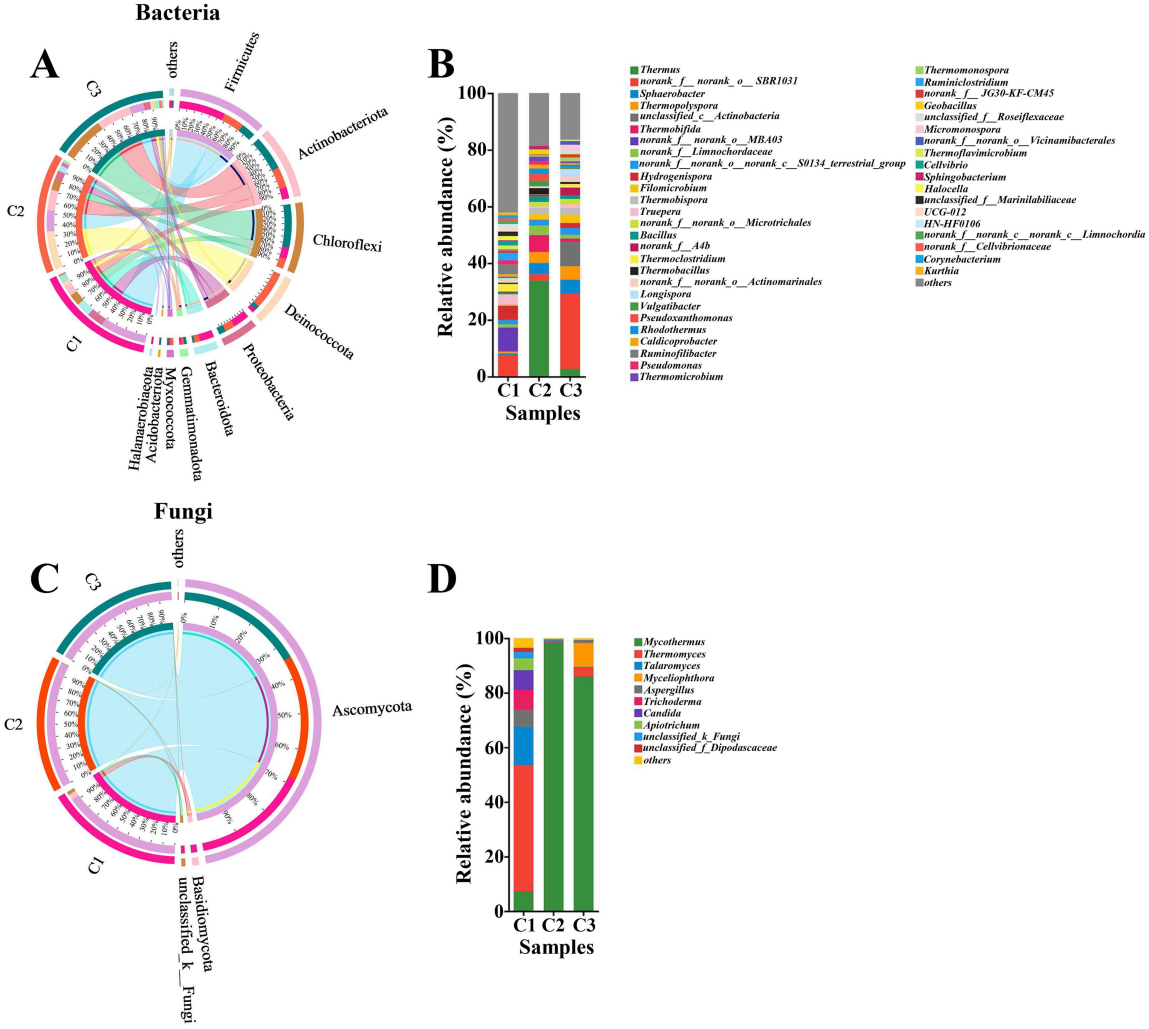
Circos diagram at phylum level and relative abundance at genus level of bacteria (A,B) and fungi (C,D) during the composting of tunnel composting technology. Phyla and genera with relative abundance below 1% were combined together and indicated as “others”.

As composting proceeded, the succession of microbial communities also changed obviously. Based on a sequence similarity of >97%, 1,538 and 383 OTUs, 27 and 7 phyla, 73 and 21 classes, 191 and 47 orders, 322 and 94 families, 641 and 153 genera, 1,027 and 240 species of bacteria and fungi were, respectively, identified by metagenomic analysis. This result showed that the abundance of bacteria was significantly higher than that of fungi and similar results were obtained by Thai et al. (2022) during preparation of *A. bisporus* compost substrate. As shown in Figure 2A, the major six bacterial phyla, namely Firmicutes (13–41%), Actinobacteriota (9.9–31%), Chloroffexi (8.9–39%), Deinococcota (3.4–34%), Proteobacteria (6.0–15%) and Bacteroidota (1.5–13%) were observed in all phases. Moreover, the relative abundance of the major bacterial phyla obviously varied in different phases. In the initial phase (C1), Firmicutes (41%), Proteobacteria (15%), Bacteroidota (13%), Chloroffexi (10%), and Actinobacteriota (9.9%) were the most abundant, followed by Deinococcota (3.4%), Gemmatimonadota (1.9%), Halanaerobiaeota (1.5%), and Myxococcota (1.1%) among which *Hydrogenispora* (5.03%), *Ruminofilibacter* (3.50%), *Truepera* (3.28%), *Thermoclostridiumm* (2.71%), and *Ruminiclostridium* (2.53%) were the top five bacterial genera (Figure 2B). These results were significantly different from previous study, in which Proteobacteria was the dominant phylum at the early stage (Kong et al., 2020). This result indicated that different raw materials of composting resulted in different microbial communities. Additionally, different composting phases have different dominant microorganisms. Interestingly, the proportion of Deinococcota increased by 33.6% and became the most abundant phylum in C2 phase. Firmicutes and Actinobacteriota were the other two abundant bacterial phyla in the thermophilic stage. Firmicutes was a common group of bacteria in composting and they could survive normally under high temperature (Guo et al., 2021; Sangeetha et al., 2017; Yang et al., 2020). The higher abundance of Firmicutes suggested the faster degradation rate of cellulose in C2 phase. Actinobacteriota also had a vital role in biodegradation of organic matter (Wei et al., 2018). The abundance of Actinobacteriota had a significant positive correlation with cellulolytic enzyme activities, which also explained the rapid increase in the rate of cellulose degradation in C2 phase. *Thermus* (33.81%), *Thermobifida* (5.84%), *Sphaerobacter* (3.96%), and *Thermopolyspora* (3.92%) became the predominant bacterial genera in C2 phase. The abundance of Actinobacteriota phylum continuously increased until the end of composting. Firmicutes maintained a relatively stable range (19–13%) from C2 to C3. Chloroffexi reached the highest relative abundant level of 39% in C3 phase. Proteobacteria softly decreased from 8.8 to 6.0% in C3 phase. Proteobacteria were detected in large amounts in *A. bisporus* substrate composting which were known to play an important role in biomass degradation (Székely et al., 2009; Vieira and Pecchia, 2022). The decline of Proteobacteria may be unfavorable to the transformation of NO_2_-N to NO_3_-N (Ren et al., 2016; Zhang et al., 2012). Unclassified_c__Actinobacteria, *Sphaerobacter*, *Thermopolyspora* and *Filomicrobium* took up 8.69, 4.83, 4.79 and 2.96%, which were the top four predominant bacterial genera in C3 phase. However, the microbial communities were dominated by the *Thermopolyspora*, *Microbispora*, *Chelatococcus*, and *Pseudoxanthomonas* at the end of Phase II of *A. bisporus* compost (Shamugam and Kertesz, 2023).

As shown in Figure 2C, the major two fungal phyla, namely Ascomycota (93–99%), Basidiomycota (0.09–4.5%) were appeared in all phases. Yang et al. (2022) also found that the fungal community was dominated by Ascomycota (90.41–94.80%) and Basidiomycota (2.92–8.96%) during short-term composting for the substrate for oyster mushrooms. Ascomycota and Basidiomycota were common groups of fungi and widely distributed during composting based on various raw materials. In particular, Ascomycota played a great role in cellulose degradation (Guo et al., 2021; Zhang et al., 2011). When the composting entered thermophilic phase (C2), the proportion of Ascomycota increased by 6%, which showed that Ascomycota could adapt well to high temperature environment (Guo et al., 2021). The relative abundance of Ascomycota remained roughly the same from C2 to C3. *Thermomyces* (46.32%), *Talaromyces* (14.09%), *Trichoderma* (7.31%), *Candida* (7.32%) and *Mycothermus* (7.19%) were the dominant fungal genera in C1 phase. *Trichoderma* were also present in compost raw materials of *A. bisporus* and act as a biological control agent against certain pathogens (Pandin et al., 2018; Vieira and Pecchia, 2022). *Mycothermus* sharply increased to 98.36% and become the only dominant fungal genus, while *Thermomyces*, *Talaromyces* and *Trichoderma* were not detected or less than 0.5% in the thermophilic phase (C2). *Mycothermus* (85.94%) underwent a slight decline from C2 to C3, while it was still the most abundant genus (Figure 2D). However, the fungal community was entirely dominated by *Mycothermus thermophiles* in Phase II of *A. bisporus* compost, which stimulated growth of the button mushroom mycelium (Coello-Castillo et al., 2009; Thai et al., 2022).

In addition, LEfSe was conducted to identify microbial indicator groups in different composting phases (Supplementary Figure S1). In the initial phase, the indicator groups for bacteria were Firmicutes and Bacteroidia. Thermus and Thermobifida were the indicator groups in the thermophilic phase. Longispora and unclassified_ Actinobacteria were identified as the indicators in the maturation phase. Chytridiomycota was the fungal indicator group in C1 phase. There were 4 and 2 indicator groups in C2 and C3, respectively.

### 3.5 Network correlation analysis

**Figure 3:**
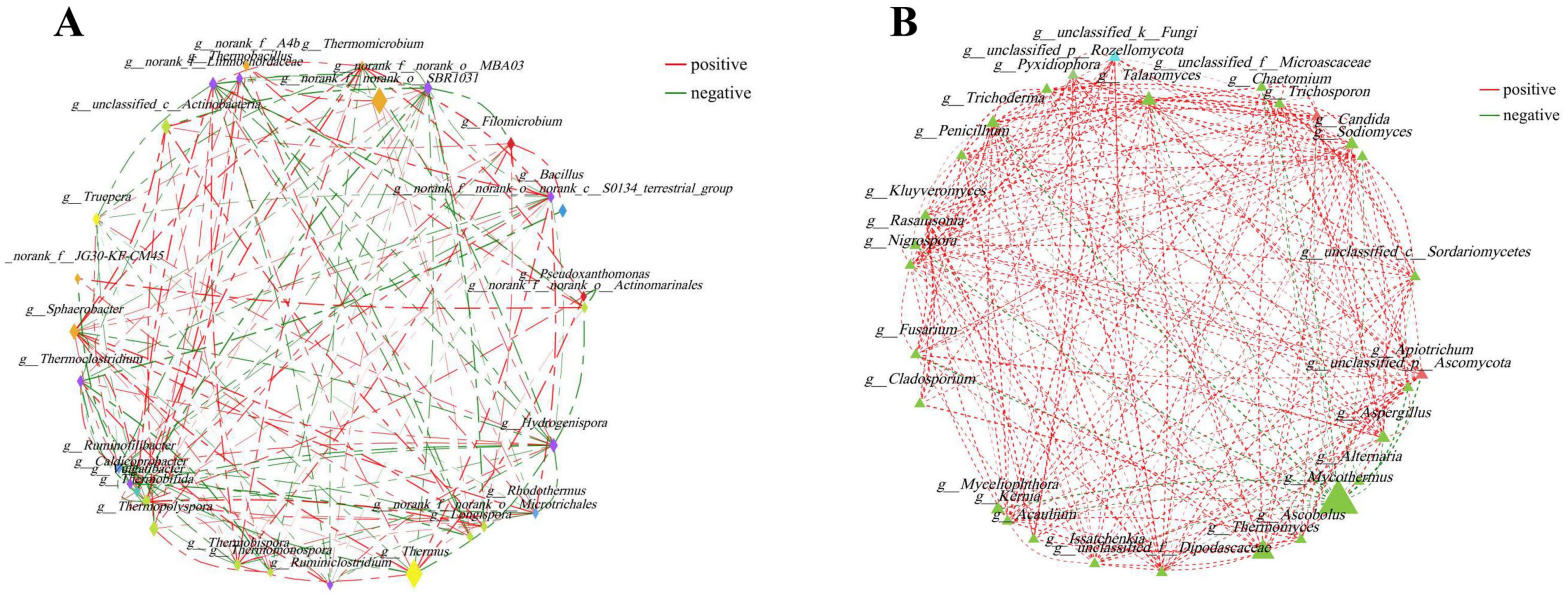
Network analysis was applied to bacteria **(A)** and fungi **(B)** during the composting of tunnel composting technology. The size of the node was proportional to the genera abundance. Node color corresponded to phylum taxonomic classification. Edge color represented positive (red) and negative (green) correlations, and the edge thickness was equivalent to the correlation values.

The potential interrelationships between microorganisms were evaluated by network analysis. The network of top 30 bacterial genera comprised 29 nodes and 152 edges (including 83 positive, 69 negative). Moreover, the top five predominant bacterial genera were *Thermus*, *norank_f__norank_o__SBR1031*, *Sphaerobacter*, *Thermopolyspora* and *Hydrogenispora*. They had 10 (including 5 positive, 5 negative), 5 (including 3 positive, 2 negative), 12 (including 7 positive, 5 negative), 7 (including 5 positive, 2 negative), and 12 (including 5 positive, 7 negative) edges, respectively (Figure 3A). The network of the top 30 fungal genera comprised 29 nodes and 243 edges (including 231 positive, 12 negative). *Mycothermus*, *Thermomyces*, *Talaromyces*, *Candida* and *Trichoderma* were the top five fungal genera. They had 13 (including 4 positive, 9 negative), 12 (including 11 positive, 1 negative), 18 (all positive), 14 (all positive), and 18 (including 17 positive, 1 negative) edges, respectively (Figure 3B).

### 3.6 Alpha and beta diversity of microbial communities

Alpha diversity analysis is mainly used to evaluate the richness and diversity of microbial communities in different samples through multiple diversity index (Rogers et al., 2016). The coverage of each sample was greater than 0.99, indicating that the sequencing data included most bacteria and fungi. The bacterial Chao1 indices in C1, C2, and C3 phases were 1476.25, 667.74, and 657.38, and the fungal Chao1 indices were 268.58, 61.70, and 77.33, respectively. The bacterial Chao1 indices were higher than that of fungi, which reflected that there were more OTUs in bacterial communities and the richness of bacterial communities was higher. The Chao1 indices showed the greatest change from C1 to C2, which indicated that the microbial communities changed significantly during this period. Kertesz and Thai (2018) also found that the most dramatic changes in microbial populations in compost of *A. bisporus* occurred during the initial period and phase I (Kertesz and Thai, 2018). The ratios of Shannon index/Simpson index of bacteria were significantly higher than that of fungi, which suggested that the composition of bacterial community was more complex. With the composting, Shannon index decreased first and then increased following the order: C1 > C3 > C2 (Supplementary Table S2). This change indicated that the microbial communities decreased in the thermophilic stage (C2), which was due to the high temperature killing many miscellaneous bacteria.

To verify the temporal distribution differences of the microbial communities, the PCoA analysis was performed on the bacterial and fungal communities (Supplementary Figure S2). Bacterial community structure was clearly divided into three groups: C1, C2, and C3 comprised independent groups. This result indicated that the bacterial community structure had a significant temporal succession pattern throughout composting process (Meng et al., 2019). However, the fungal communities of C2 and C3 were well clustered together and separated from C1, showing that the fungal community structure gradually stabilized (Zhang et al., 2020). The results of the NMDS analysis were consistent with the PCoA. The NMDS showed that the microbial community structures of bacteria were significantly different between the composting phases (Supplementary Figure S3).

### 3.7 Relationships analysis

**Figure 4:**
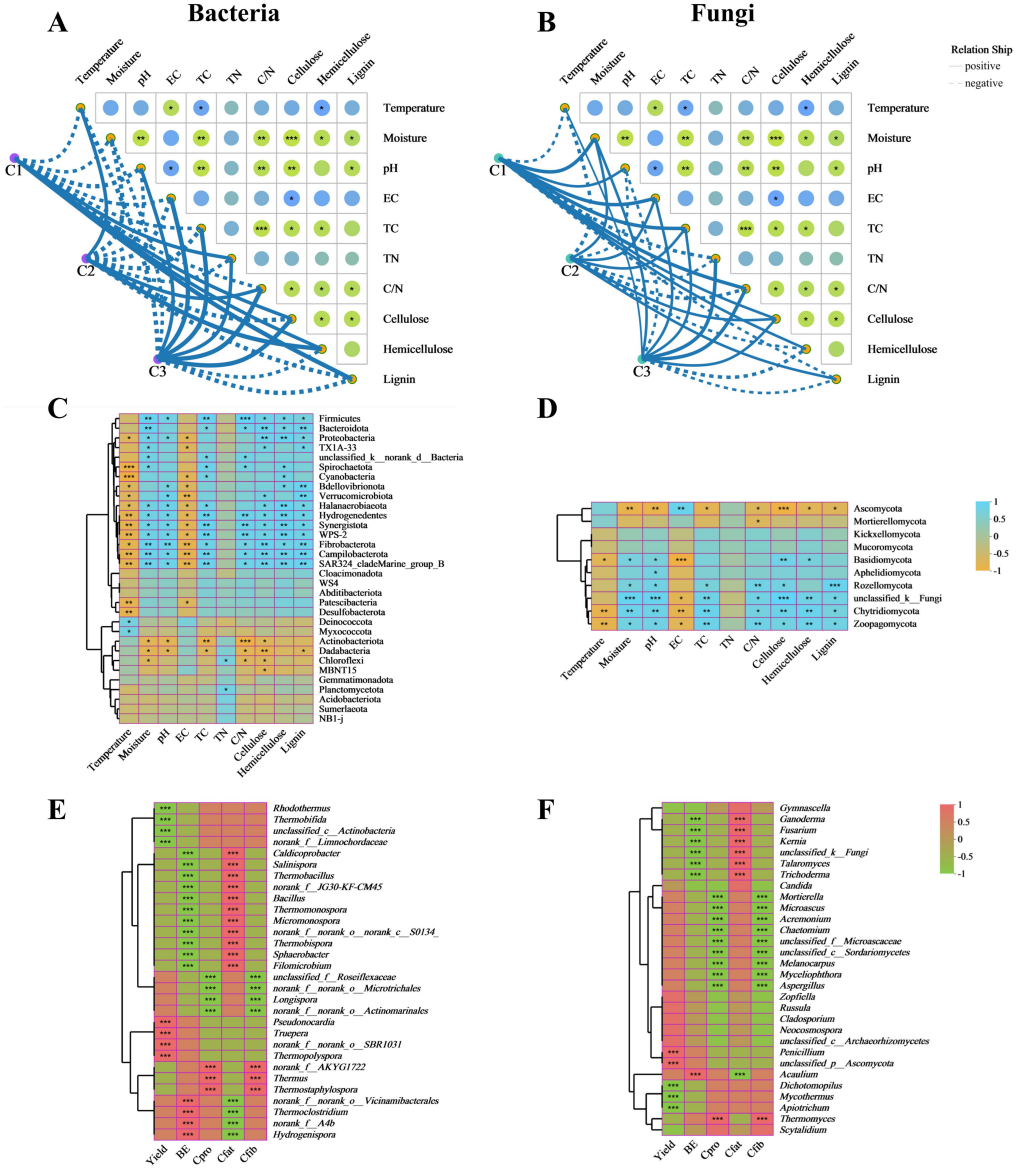
Relationships analysis of microbial communities with physicochemical, agronomic and nutritional properties. Associations of physicochemical properties with bacterial communities (A) and fungal communities (B) during different composting phases were analyzed by the Mantel tests. Correlation heat map of physicochemical properties and microbial communities at phylum level of bacteria (C) and fungi (D). The correlation heatmap of the bacterial (E) and fungal (F) top 30 genera with agronomic and nutritional properties of *A. subrufescens*. TC, total carbon; TN, total nitrogen; C/N, C/N ratio; BE, biological efficiency; Cpro, crude protein content; Cfat, crude fat content; Cfib, crude ffber content. *0.01 ≤ *p* < 0.05, **0.001 ≤ *p* < 0.01, ***p < 0.001.

The Mantel test was used to determine that the physicochemical properties of composting substrates were significantly associated with microbial communities in different composting phases (Figures 4A,B). The results showed that TN and the lignocellulose contents were significantly positively correlated with the bacterial community in the initial phase (C1). There were significant positive correlations between bacterial community and temperature, C/N ratio, the content of hemicellulose in C2 phase. Moreover, temperature was the key influencing factor in the thermophilic phase (temperature > hemicellulose > C/N ratio). Microbial metabolism affected the change of temperature, and temperature also drived the succession of microbial community (Yang et al., 2020). The bacterial abundance of C3 phase showed the significantly positively correlated with the pH, EC, TC, TN, and C/N ratio (Figure 4A). Therefore, the succession of bacterial community was affected by different physicochemical properties in different composting stages (Guo et al., 2021; Kong et al., 2020). Based on fungal community, the main influencing factors had not changed in C1 phase. When the composting entered the thermophilic phase, moisture, pH, EC, TC, cellulose and lignin became the main environmental factors (moisture > EC > TC > cellulose > lignin > pH). However, only TN was not significantly correlated with the fungal community (Figure 4B). Similar result had been obtained by Xu et al. (2023). As composting proceeded, the physicochemical properties including temperature, moisture, EC, TC, TN, C/N ratio and cellulose played positive roles in composting maturity (C3).

The correlation heat map showed that TN had a minor effect on the relative abundance of bacterial phyla. Only Chloroflexi and Planctomycetota appeared to show significant positive relationships with TN. TC and C/N ratio revealed significant positive correlations with Bacteroidota, Spirochaetota and Fibrobacterota and extremely significant positive correlations with Firmicutes, Hydrogenedentes and Synergistota. Cellulose, hemicellulose and lignin were significantly positively correlated with Firmicutes and extremely significantly correlated with Campilobacterota. Moisture and pH demonstrated significant positive correlations with Proteobacteria, Halanaerobiaeota, Hydrogenedentes, and Synergistota and extremely significant positive correlations with Fibrobacterota. Temperature and EC were significantly negatively correlated with Cyanobacteria, Bdellovibrionota, Verrucomicrobiota, Halanaerobiaeota, Hydrogenedentes, Synergistota, Fibrobacterota, Campilobacterota, and Patescibacteria (Figure 4C). Based on fungal communities, TN was also the least effective environmental factor. TC and C/N ratio showed significant positive correlations with Rozellomycota, Chytridiomycota, and Zoopagomycota. The lignocellulose contents were significantly positively correlated with Chytridiomycota and Zoopagomycota and negatively correlated with Ascomycota. Moisture and pH appeared to show significant positive relationships with Basidiomycota, Rozellomycota, Chytridiomycota, and Zoopagomycota and extremely significant negative correlations with Ascomycota. Temperature and EC revealed significant negative correlations with Basidiomycota, Chytridiomycota, and Zoopagomycota (Figure 4D).

The microbial communities also play significant roles in the yield and quality of edible fungi (Yang et al., 2022). As shown in Figure 4E, Pearson correlation between the main bacterial genera and agronomic and nutritional properties indicated that *Pseudonocardia*, *Truepera*, and *Thermopolyspora* were positively correlated and significant with yield at C3 stage. *Thermoclostridium* and *Hydrogenispora* were positively correlated and significant with BE while negatively correlated with crude fat content. *Caldicoprobacter*, *Salinispora*, *Thermobacillus*, *Bacillus*, *Thermomonospora*, *Micromonospora*, *Thermobispora*, *Sphaerobacter*, and *Filomicrobium* represented significant positive correlations with crude fat content. *Bacillus* appeared to show significant positive relationships with yield and BE in composting substrates of oyster mushroom cultivation (Wei et al., 2024). *Thermus* and *Thermostaphylospora* showed significant positive correlations with crude protein content and crude fiber content. Based on the above results, it was concluded that agronomic and nutritional properties were affected by different microbial communities. Therefore, the higher abundance of bacterial community was more beneficial to the growth of edible fungi. Based on fungal community, *Thermomyces*, the predominant genus in C1 phase, represented significant positive correlations with crude protein content and crude fiber content. *Penicillium* demonstrated significant positive correlation with yield. *Acaulium* appeared to show significant positive relationship with BE (Figure 4F). Previous studies reported that *Acaulium* were involved in the degradation of lignocellulose (Zhang et al., 2020). Similar to bacterial communities, more fungal genera represented significant positive correlations with crude fat content.

### 3.8 Functional characteristics of bacterial community

**Figure 5:**
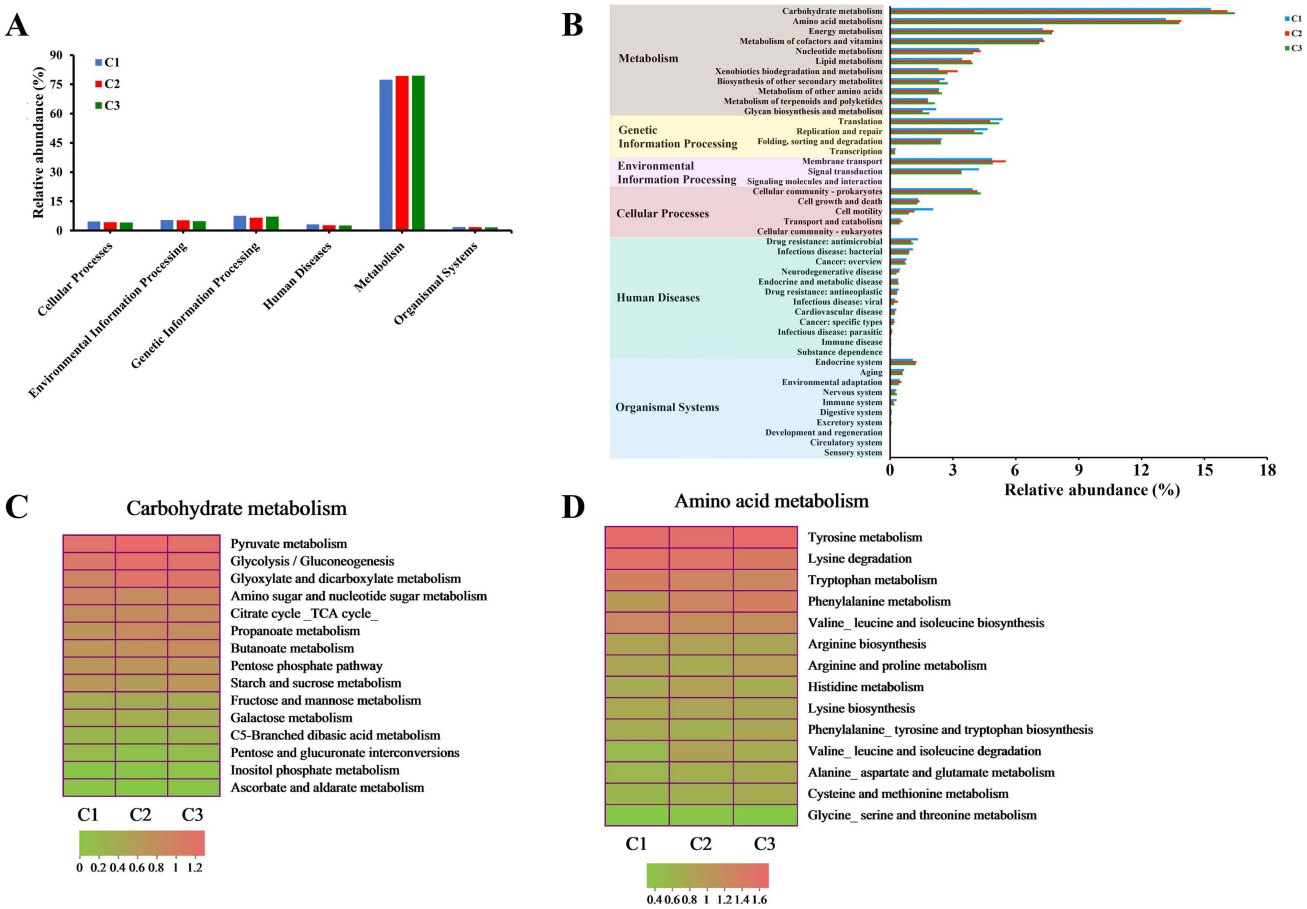
Variation of bacterial functional profiles during different composting phases. (A,B) The level 1 and level 2 KEGG ortholog function annotation, respectively; (C) level 3 function categories of carbohydrate metabolism; (D) level 3 function categories of amino acid metabolism.

In order to further investigate the potential functions and physiological capabilities of bacterial communities in different composting phases of tunnel composting technology, KEGG pathway annotation of the predicted gene sequences obtained from 9 substrate samples was performed based on the Kyoto Encyclopedia of Genes and Genomes (KEGG) pathway database.^6^ In total, 6 functional groups (pathway level 1) were identified: metabolism (77.37–79.41%), genetic information processing (6.69–7.58%), environmental information processing (4.87–5.41%), cellular processes (4.11–4.66%), human diseases (2.66–3.19%), and organismal systems (1.70–1.78%) (Figure 5A). Moreover, 11 pathways (level 2) of metabolism, 4 pathways of genetic information processing, 3 pathways of environmental information processing, 5 pathways of cellular processes, 12 pathways of human diseases, 10 pathways of organismal systems were obtained (Figure 5B). 389 KEGG orthologs at level 3 were detected. The metabolisms of carbohydrate, amino acid and energy were the three main pathways, which was consistent with previous result obtained by Liu et al. (2022) during *Pleurotus ostreatus* mushroom cropping on a short composting substrate. As the composting continued, their relative abundance increased in the thermophilic phase (C2). In addition, the relative abundance of carbohydrate metabolism continuously increased and reached the highest level in the maturation phase (C3). It indicated that carbohydrate metabolism was more active and continuously promoted the decomposition of lignocellulose during the whole composting process. Amino acids were the main energy sources for bacterial metabolism (López-González et al., 2015). The higher metabolic intensity led to the highest relative abundance of amino acid metabolism in the thermophilic phase (C2), which was beneficial to promote the synthesis of humic substance (Wu et al., 2017). The most enriched pathways, ranked from highest to lowest level of enrichment, were pyruvate metabolism, glycolysis/gluconeogenesis and glyoxylate and dicarboxylate metabolism in carbohydrate metabolism. The relative abundance of the three pathways increased from 1.86, 1.75 and 1.57% to 2.10, 1.97 and 1.89% during the early composting process, reaching the highest level in C2 phase and remained stable level in C3 phase (Figure 5C). The top 3 most enriched pathways in amino acid metabolism were tyrosine metabolism, lysine degradation and tryptophan metabolism. Their relative abundance softly decreased with the composting time (Figure 5D).

## 4 Conclusion

As far as we know, this study firstly revealed the dynamic succession of microbial compost communities and functions, and the relationships between microbial communities and agronomic and nutritional properties during the *A. subrufescens* cultivation using tunnel composting technology. The physicochemical characteristics varied greatly in different composting phases. Moreover, the physicochemical properties significantly affected the succession of microbial communities, which further affected agronomic properties and nutritional qualities of *A. subrufescens*. The dominant microbial communities mainly included Firmicutes, Actinobacteriota, Chloroffexi, Deinococcota, Proteobacteria and Bacteroidota of bacteria, Ascomycota and Basidiomycota of fungi. Moisture, pH, TC, C/N ratio were significantly positively correlated with bacterial communities. TN was the environmental factor with the least impact on microbial communities. *Pseudonocardia*, *Truepera*, *Thermopolyspora* and *Penicillium* were positively correlated and significant with the yield of *A. subrufescens*. Carbohydrate, amino acid and energy metabolisms were the three main pathways. Overall, the findings will provide a better understanding and valuable advice for the composting process of tunnel composting technology for preparation of *A. subrufescens* cultivation substrates.

## Data Availability

The datasets presented in this study can be found in online repositories. The names of the repository/repositories and accession number(s) can be found at: https://www.ncbi.nlm.nih.gov/sra, accession numbers SRP473681 and SRP473655.

## Footnotes

^1^http://drive5.com/uparse/

^2^https://www.arb-silva.de/

^3^http://unite.ut.ee/index.php

^4^http://www.mothur.org/wiki/Schloss_SOP#Alpha_diversity

^5^https://www.i-sanger.com/

^6^http://www.genome.jp/kegg/
