# Supplementary material for: Microbial communities during the composting process of Agaricus subrufescens and their effects on mushroom agronomic and nutritional qualities
Source: Front Microbiol. 2024 Nov 15;15:1471638. doi: 10.3389/fmicb.2024.1471638 (PMC11604460; doi:10.3389/fmicb.2024.1471638)
Supplement: Supplementary file 1 [file Table_1.DOCX]

Supplementary Material

**Supplementary Table S1.** Physicochemical properties of the raw materials.

| Raw material | Moisture (%) | pH | EC (mS/cm) | Total carbon (%) | Total nitrogen (%) | Cellulose (%) | Hemicellulose (%) | Lignin (%) |
| --- | --- | --- | --- | --- | --- | --- | --- | --- |
| cow manure | 13.79 ± 0.88a | 8.69 ± 0.21a | 5.83 ±0.77a | 52.85 ± 1.73c | 2.42 ± 0.16a | 18.67 ± 0.56c | 12.18 ± 0.72c | 26.39 ±2.01b |
| corncob | 8.35 ± 0.41b | 4.87 ± 1.09c | 1.48 ±0.25b | 53.79 ± 1.34b | 0.59 ± 0.04c | 46.12 ± 0.77a | 30.49 ± 1.52a | 21.07 ± 1.33c |
| wheat straw | 6.71 ± 0.73c | 5.36 ± 2.33b | 1.27 ±0.09c | 54.61 ± 1.52a | 0.67 ± 0.08b | 37.35 ± 0.64b | 14.84 ± 1.05b | 35.82 ± 4.51a |

All the data were expressed as means ± SD (n = 3). Different lowercase letters indicate significant differences (*P* < 0.05).

**Supplementary Table S2.** Alpha diversity index statistics.

All the data were expressed as means ± SD (n = 3). Different lowercase letters indicate significant differences (*P* < 0.05).

| Microbial type | Sample | Coverage | Shannon | Simpson | ACE | Chao 1 |
| --- | --- | --- | --- | --- | --- | --- |
| Bacteria | C1 | 0.99 ± 0.00b | 5.35 ± 0.41a | 0.02 ± 0.00c | 1475.56 ± 89.61a | 1476.25 ± 135.87a |
|  | C2 | 0.99 ± 0.00a | 3.46 ± 0.19b | 0.15 ± 0.07a | 651.01 ± 55.83b | 667.74 ± 42.95b |
|  | C3 | 0.99 ± 0.00a | 3.66 ± 0.26b | 0.09 ± 0.03b | 636.72 ± 23.69b | 657.38 ± 72.61b |
| Fungi | C1 | 0.99 ± 0.00a | 2.41 ± 0.67a | 0.23 ± 0.12c | 272.50 ± 64.29a | 268.58 ± 25.23a |
|  | C2 | 0.99 ± 0.00a | 0.17 ± 0.09c | 0.96 ± 0.25a | 62.04 ± 11.75c | 61.70 ± 11.39c |
|  | C3 | 0.99 ± 0.00a | 0.46 ± 0.18b | 0.79 ± 0.19b | 79.78 ± 18.52b | 77.33 ± 15.32b |

**
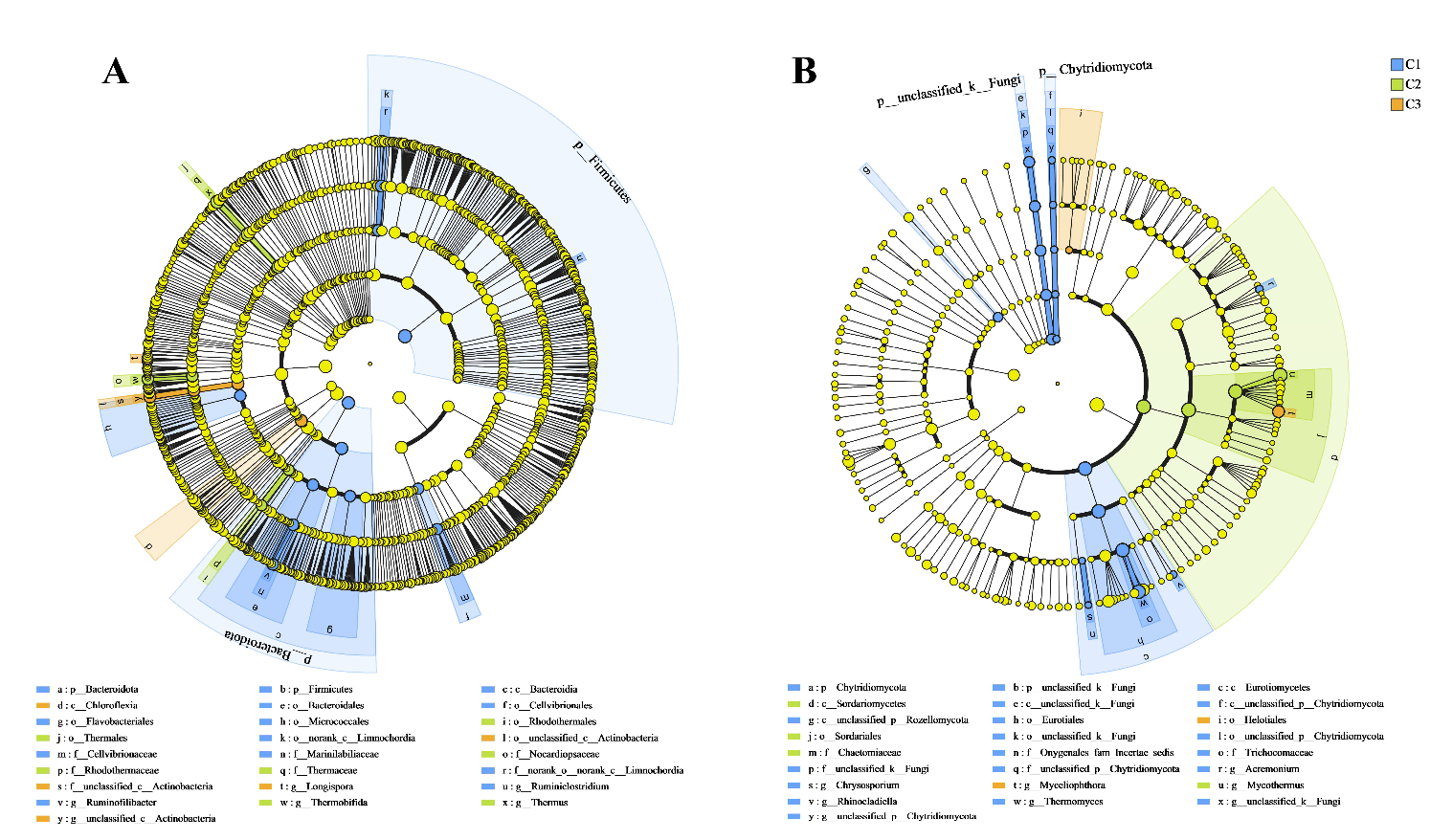
**

**Supplementary Figure 1.** LEfSe analysis of bacterial communities (A) and fungal communities (B) during composting (*P* < 0.05, LDA scores > 4.0). LEfSe, linear discriminant effect size analysis.


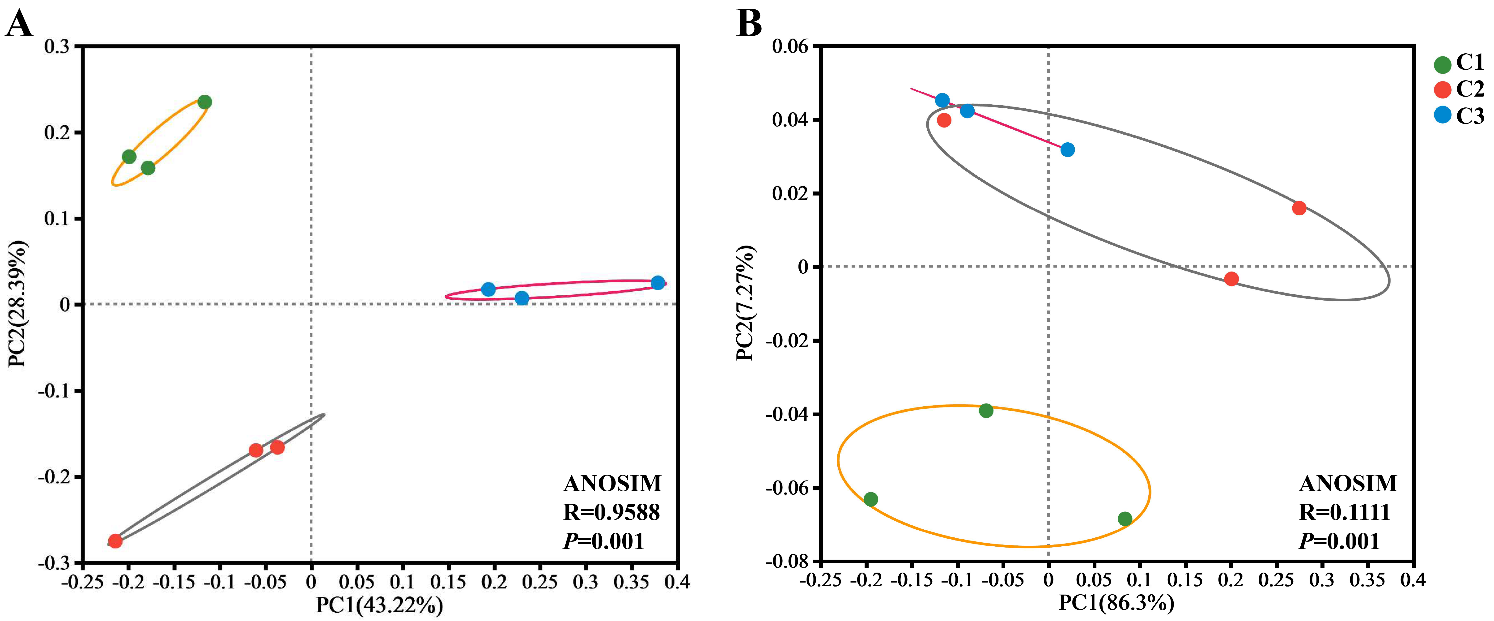


**Supplementary Figure 2.** PCoA of bacterial (A) and fungal (B) communities in different composting substrates at OUT level.


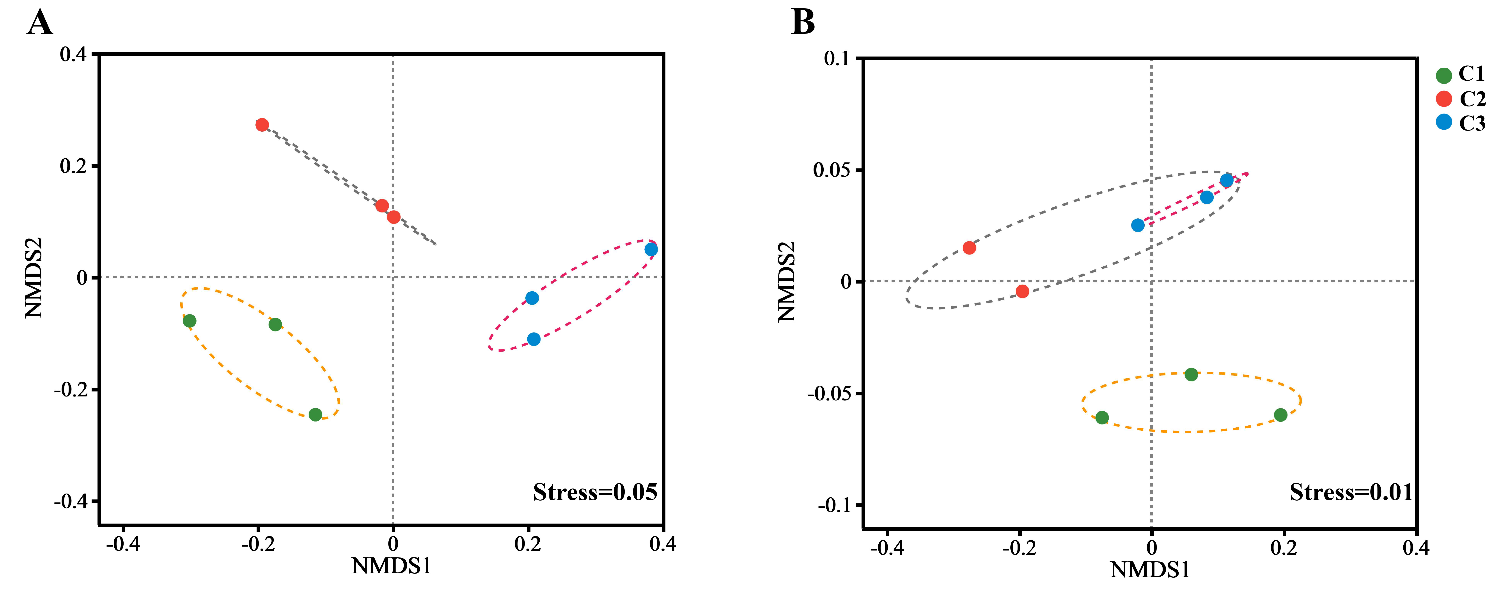


**Supplementary Figure 3.** Bray-Curtis-based non-metric multidimensional scaling (NMDS) analysis of bacterial (A) an fungal (B) community structures.
